# Hormones as go‐betweens in plant microbiome assembly

**DOI:** 10.1111/tpj.15135

**Published:** 2021-01-25

**Authors:** Ruth Eichmann, Luke Richards, Patrick Schäfer

**Affiliations:** ^1^ Institute of Molecular Botany Ulm University Ulm 89069 Germany; ^2^ School of Life Sciences University of Warwick Coventry CV4 7AL UK

**Keywords:** endosphere, microbiota, plant development, plant evolution, rhizosphere, symbiosis

## Abstract

The interaction of plants with complex microbial communities is the result of co‐evolution over millions of years and contributed to plant transition and adaptation to land. The ability of plants to be an essential part of complex and highly dynamic ecosystems is dependent on their interaction with diverse microbial communities. Plant microbiota can support, and even enable, the diverse functions of plants and are crucial in sustaining plant fitness under often rapidly changing environments. The composition and diversity of microbiota differs between plant and soil compartments. It indicates that microbial communities in these compartments are not static but are adjusted by the environment as well as inter‐microbial and plant–microbe communication. Hormones take a crucial role in contributing to the assembly of plant microbiomes, and plants and microbes often employ the same hormones with completely different intentions. Here, the function of hormones as go‐betweens between plants and microbes to influence the shape of plant microbial communities is discussed. The versatility of plant and microbe‐derived hormones essentially contributes to the creation of habitats that are the origin of diversity and, thus, multifunctionality of plants, their microbiota and ultimately ecosystems.

## Significance Statement

Plants and all their tissues are inhabited by, surrounded by and, thus, in interactions with complex microbial communities. Plant hormones drive the establishment of these communities, termed microbiota, which add a diversity of functions to plants that are crucial for plant fitness under changing environments since the time of land colonisation.

## INTRODUCTION

**Figure 1:**
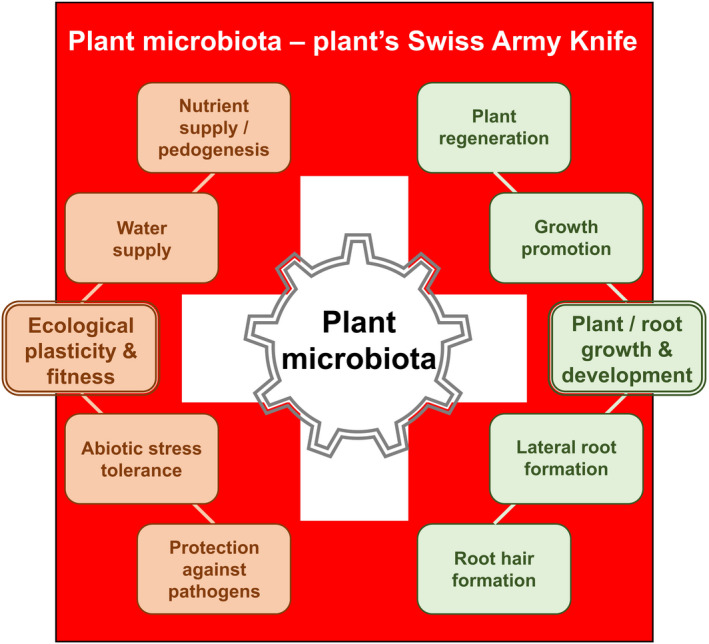
Plant microbiota are a plant’s Swiss Army Knife. To access the benefits of the microbiota, plants need to have some control over their assembly, and hormones play a crucial role therein. The benefits can be divided into two branches: (i) enhanced ecological plasticity and fitness based on improved nutrient and water supply as well as protection against biotic and abiotic stress and (ii) optimised plant/root growth and development due to microbial support in plant regeneration, growth and the formation of lateral roots and root hairs.

**Figure 2:**
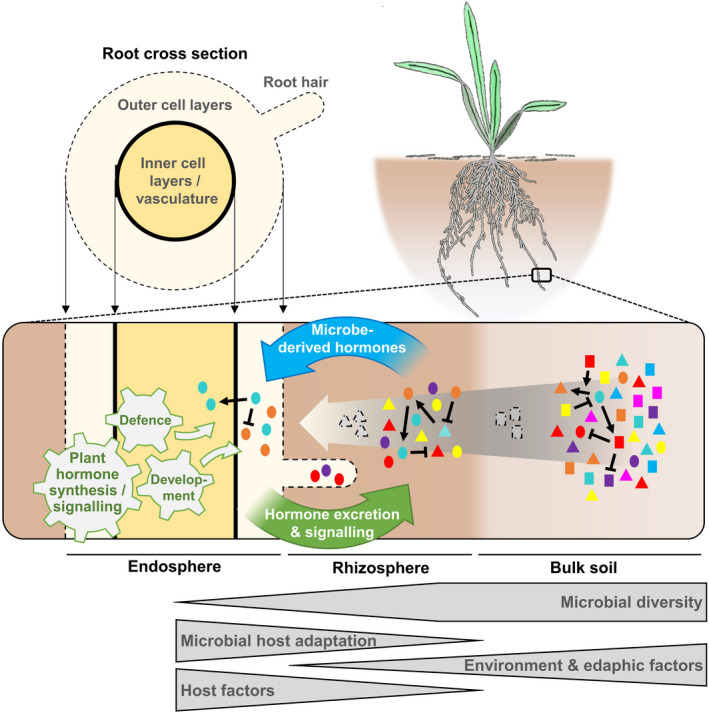
Hormone function in the assembly of microbiota. The ability of soil microbes to colonise the rhizosphere or endosphere is dependent on their degree of specialisation (indicated by grey arrow in the inner scheme). While the abiotic environment and edaphic factors (e.g. soil properties) affect the composition of microbial communities in bulk soil, microbe‐derived and plant‐excreted hormones contribute to their assembly in the plant rhizosphere. Hormone‐dependent developmental and defence‐related processes inside the plant shape the endosphere community. Microbe–microbe interactions contribute to the assembly of microbiomes across all habitats (indicated by ↑ and ↓). While, in general, microbial diversity is found to be lower in rhizosphere versus bulk soil, metatranscriptome analyses revealed similar diversities in both compartments but differences in microbial composition (Turner *et al*., 2013b). Due to further evolution‐driven microbial host adaptation processes and host factors, microbial diversity is lower in the endosphere and only highly host‐adapted microbes are able to colonise outer and/or inner root layers (incl. vasculature).

Plants, like all multicellular organisms, do not live in a sterile environment. The in‐ and outsides of plants are populated by specific and often selectively assembled microbial communities, called microbiota (Bulgarelli *et al*., 2013; Goodrich *et al*., 2016; Orozco‐Mosqueda *et al*., 2018). These microbial communities can be vast in the range of species/taxa (diversity) and number of individuals present (abundance). The sheer quantity of microbial species found to be associated with plant tissues (e.g. leaves, roots) together with the genetic and functional diversity of those microbial communities has given rise to the term microbiome (the collective genomes of an organism’s microbiota) (Handelsman *et al*., 2007). The soil biota represents the origin of plant‐associated microbiomes. Microbiome compositions differ between plant organs and are therefore defined, for example, as phyllosphere or rhizosphere microbiomes for communities attached to the outside of leaves and roots, respectively, or endosphere microbiomes for communities found inside plant tissues (Knief *et al*., 2012; Bulgarelli *et al*., 2013; Turner *et al*., 2013a; Berg *et al*., 2014; Bai *et al*., 2015; Cregger *et al*., 2018). The best‐characterised members of plant microbiomes are bacteria and fungi. They can live in neutral, beneficial or pathogenic interaction within or outside of the plant (Raaijmakers *et al*., 2009; Turner *et al*., 2013a). Unsurprisingly, in addition to environmental niche effects and edaphic factors, plants have evolved mechanisms to shape such communities, benefit from and even exploit microbiomes as a huge genetic resource to expand their ability to cope with changing environmental conditions (Bulgarelli *et al*., 2012; Lundberg *et al*., 2012; Philippot *et al*., 2013; Reinhold‐Hurek *et al*., 2015; Liu *et al*., 2019; Brown *et al*., 2020). The ability of plant roots to modify microbial communities is strongest in the endosphere but can reach well beyond the rhizosphere. Under leaf pathogen attacks, for instance, plant roots excrete metabolites to change the composition of the soil biota as a strategy to recruit beneficial microbes that activate effective defence against the leaf invaders (Lakshmanan *et al*., 2012; Chaparro *et al*., 2013; Berendsen *et al*., 2018; Stringlis *et al*., 2018). Consequently, as for humans, the plant microbiome has been referred to as the extended or secondary genome of plants, as it encodes huge numbers of genes and may thus provide additional genetic and functional diversity to the host (Grice and Segre, 2012; Berendsen *et al*., 2012; Mendes *et al*., 2013; Turner *et al*., 2013a). The versatile effects of the plant microbiota can be roughly divided into two fractions (Figure 1) (Mendes *et al*., 2013). Firstly, microbial communities can improve the environmental adaptability and fitness of plants by contributing to plant protection against abiotic stress (e.g. drought) or pathogens (incl. herbivores) as well as by fostering nutrient and water supply. The latter also involves pedogenetic effects of microbes. Secondly, microbes support plant and root system architecture. In addition to stimulating lateral root growth and root hair formation, which further improves water and nutrient accessibility, microbes can promote growth and plant regeneration. Together, this indicates the ability of microbes to steer plant intrinsic and extrinsic processes that support the sessile nature of plants and help them to grow and reproduce under changing environments (Figure 1). The questions arise how and to what extent can plants shape microbiomes to their own benefit. Within the colonised zones, plants and their microbial communities strongly influence each other within the given environmental conditions (Fitzpatrick *et al*., 2020). Various mechanisms describe how plants communicate with and, thus, change their living environment. Chemical cues contribute strongly to antagonistic (e.g. through the production of antimicrobial metabolites) or mutually supportive (e.g. through the production of probiotics) microbe–microbe interactions (Bulgarelli *et al*., 2015; Hacquard *et al*., 2015; Pieterse *et al*., 2016; Hassani *et al*., 2018). For instance, volatiles have been very versatile for plants and even plant communities to protect them against insects (Sharifi *et al*., 2018; Hammerbacher *et al*., 2019). Primary and secondary metabolites produced and exuded by the plant can selectively attract or repel members of microbial communities (Leach *et al*., 2017; Nobori *et al*., 2018). Vice versa, microbial metabolites can alter plant development and responses to environmental cues, often contributing to increased plant health and fitness (Strehmel *et al*., 2014; Venturi and Keel, 2016; Sasse *et al*., 2018; Fitzpatrick *et al*., 2020; O’Banion *et al*., 2020). In this whole process of plant–microbiota interactions, plant hormones take a central role. Hormones are chemical messengers that are involved in cellular and physiological processes in an endocrine (function distant to biosynthesis site) or paracrine (function in cells adjacent to biosynthesis site) manner. In addition to edaphic factors, hormones are part of host factors (e.g. nutrients, signalling processes) and microbial adaptation processes to their hosts. In this way, hormones can contribute to the microbial diversity in the endosphere and different root compartments by regulating plant defence and development, or in the rhizosphere by direct or indirect activities of excreted hormones on microbes (Figure 2). This review presents our current knowledge of hormone effects on the composition and diversity of plant microbiomes. Taking three perspectives, we will describe to what extent (i) hormone signalling inside plants, (ii) hormones excreted by plants and (iii) microbe‐derived hormones affect microbial communities (Figure 2). Taking different perspectives also allows us to understand that plants and microbes often employ the same hormones for completely different purposes. We introduce the function of hormones as common chemical language, whose purposeful utilisation by plants and microbes characterises them as versatile go‐betweens to establish species‐rich and functionally diverse biocenoses.

## THE EFFECTS OF HORMONE SIGNALLING INSIDE PLANTS ON PLANT MICROBIOMES

To comprehend hormone function in shaping microbiomes requires looking back to the origin and evolution of plant–microbe interactions. Plants started to colonise land at the mid‐late Ordovician period (470–443 million years ago [mya]). Terrestrial colonisation involved contending with a range of stresses and nutrient acquisition literally without a root system. Complex interactions between hormone signalling pathways have been posited as key for dealing with and fine‐tuning responses to combined biotic and abiotic stresses whilst also limiting growth trade‐offs (Yasuda *et al*., 2008; Mosher *et al*., 2010; Vos *et al*., 2015). For more details we refer to excellent reviews (Pieterse *et al*., 2009; Berens *et al*., 2017). Regulatory networks of hormones – specifically auxin, abscisic acid (ABA), cytokinins (CKs), gibberellic acid (GA), jasmonic acid (JA), salicylic acid (SA) and strigolactones (SL) – are conserved across embryophyte lineages, whose common ancestors were some of the earliest colonisers of land (Wang *et al*., 2015). GA perception and signalling, for instance, diversified as part of early land plant evolution and their adaptation to the environment (Yasumura *et al*., 2007). In this process of plant transition and adaptation to land, beneficial symbionts played some important role. Fossil records revealed the presence of plant symbioses since the Silu‐Devonian period (443–419 mya) (Remy *et al*., 1994; Martin *et al*., 2017). The presence of hormone signalling in early plants and the functional involvement of hormone networks in the outcome of plant–microbe interactions readily positions hormones as tools to be co‐opted for the regulation of plant symbioses and microbiome assembly. In addition to protection against environmental cues, an eminent task of beneficial symbionts was to serve plants in nutrient and water supply, especially under the fluctuating Silu‐Devonian climate (Selosse and Tacon, 1998).

The importance of symbionts for ecological plasticity of plants is indicated by the ability of plants to shape the microbiome to some extent (Bulgarelli *et al*., 2012; Lundberg *et al*., 2012; Yu and Hochholdinger, 2018). This suggests the existence and evolution of heritable plant traits determining the assembly of plant microbiota. Consistent with this, host phylogenetic studies have revealed an evolution‐based trajectory that can partially explain microbiota assembly (Escudero‐Martinez and Bulgarelli, 2019). Environmental stress might substantiate underlying traits as a kind of biased microbial enrichment under stress conditions. Although plant domestication can affect microbiome assembly (reviewed in Pérez‐Jaramillo *et al*., 2016), stress‐driven effects appeared to be independent of host phylogeny and domestication (Naylor *et al*., 2017; Santos‐Medellín *et al*., 2017; Fitzpatrick *et al*., 2018). This suggests a hierarchy in the mechanisms plants use to modify microbiomes and indicates the importance of stress responses (e.g. against pathogens) in microbiome assembly. Common to all those plant processes is the tight dependency on hormones.

### Hormone‐dependent plant defence processes shape endosphere microbiomes

The very effective and highly plastic way plants regulate a diversity of processes under an often rapidly changing environment is intimately linked to the function of plant hormones. Almost all hormones have been shown to participate in the plant immune system and thereby help to stop pathogen infections and to balance the interaction with beneficial symbionts (Jacobs *et al*., 2011; Pieterse *et al*., 2012; Pozo *et al*., 2015). Pathogenic microbes often use disturbances of plant hormone homeostasis to manipulate host defence responses to promote pathogenicity and virulence but also to induce cell growth and division for nutrition (Chanclud and Morel, 2016; Kunkel and Harper, 2018; Han and Kahmann, 2019). While mostly considered in bilateral plant–microbe interactions, underlying processes have fundamental impacts on the assembly and diversity of plant microbiomes. Per se, plant immune receptors do not distinguish between pathogenic and beneficial microbes but perceive them as potential intruders by conserved molecular patterns such as bacterial flagellin or fungal chitin (Jones and Dangl, 2006; Antolín‐Llovera *et al*., 2014; Teixeira *et al*., 2019; Zhou and Zhang, 2020). It is therefore not surprising that plant immunity, as a central process in the control of plant–microbe interactions, has a direct impact on the composition of microbiomes. Bilateral plant–pathogen or plant–beneficial symbiont systems have provided deep insights into the function of plant hormones as determinants of such interactions (reviewed in Robert‐Seilaniantz *et al*., 2011; Pieterse *et al*., 2012; Bürger and Chory, 2019). Reductionist plant–microbe approaches helped to define hormone functions in local and systemic plant immune responses at the site of pathogen attacks. SA, JA and ethylene (ET) are best studied for their function in plant–microbe interactions (Van Wees *et al*., 2008; Pieterse *et al*., 2009). Canonically, JA and ET signalling are involved in response to necrotrophic pathogens and specifically JA signalling in response to herbivory and wounding. SA signalling, conversely, is involved in defence responses to biotrophs (Li *et al*., 2019). In addition, those hormones were found to be instrumental in mediating systemic protection of whole plants in response to local interactions with microbes (Pieterse *et al*., 2009). SA participates in systemic acquired resistance (SAR), a defence strategy where a local pathogen attack at leaves results in plant‐wide protection against subsequent pathogen infection attempts (Kachroo *et al*., 2020). In contrast, JA and ET function in induced systemic resistance (ISR). ISR is triggered by (beneficial) rhizobacteria, which upon root interaction activate a systemic signalling process to protect the whole plant (Pieterse *et al*., 2014). Taken together, this designates hormones as part of the plant’s tool kit to keep colonisation by pathogenic and beneficial microbes under control. In this setting, plant hormones emerge as important chemical signals that, in addition to governing internal processes, are instrumental in the multidirectional communication between plants and their associated microbial communities as the most (functionally) diverse entity of their living environment (Berendsen *et al*., 2012; Lemanceau *et al*., 2017; Fitzpatrick *et al*., 2020). Information on their impact on microbiomes (particularly the endosphere), however, is only just emerging (Lebeis *et al*., 2015; Carvalhais *et al*., 2017). SA is involved in the assembly of epiphytic and endophytic root microbial communities (Kniskern *et al*., 2007; Doornbos *et al*., 2011; Lebeis *et al*., 2015). Higher alpha diversity values indicate a greater diversity and variety of species in any given microbial community. This characteristic has been shown to improve several ecosystem functions important for plant health, that is, nutrient cycling (Wagg *et al*., 2019). Alterations in alpha diversity, particularly in the endosphere and rhizosphere, could indicate an impairment in plants’ ability to recruit and maintain a typical microbial community, possibly through an inability to signal to and recognise beneficial partners in the soil or an inability to restrict colonisation by undesirable microbes. Arabidopsis treated with exogenous SA or mutants constitutively producing SA show reduced endosphere alpha diversity as well as a reduction in actinobacteria (Kniskern *et al*., 2007; Lebeis *et al*., 2015). In fact, the taxonomic profile at‐large of bacterial endosphere communities appears to be strongly driven by SA accumulation/insensitivity, as revealed by several immune signalling mutant genotypes (Lebeis *et al*., 2015). Similarly, endosphere communities of tomato (*Solanum lycopersicum*) plants constitutively degrading the ET precursor 1‐aminocyclopropane‐1‐carboxylic acid (ACC) showed a reduced alpha diversity compared to wild‐type plants (French *et al*., 2019).

Those hormone activities are not restricted to SA, JA and ET, which are historically considered as hormones that substantiate the immune response to stop pathogens. It is now obvious that hormones that were formerly considered to act only in plant development have deep‐rooted functions in shaping plant–microbe interactions and, most likely, the composition and diversity of microbiomes (Robert‐Seilaniantz *et al*., 2011; Bürger and Chory, 2019).

### Plants adjust root development to facilitate interactions with the microbiome

Plant microbiota differ between plant tissues such as flowers, leaves and roots (Ottesen *et al*., 2013). In addition, plant development affects assemblage of the rhizosphere microbiome (Chaparro *et al*., 2014). Among the plant traits that are crucial for the establishment of root‐associated microbial communities are root morphology and architecture. Root exudation differs between the different root zones, thereby attracting different microbes (Haichar *et al*., 2008; Dennis *et al*., 2010). Microbes, in turn, have specialised to colonise specific root regions and zones (Saleem *et al*., 2016). The evolution of roots therefore likely proceeded in line with the co‐evolution of plant–symbiont interactions (Selosse and Tacon, 1998; Strullu‐Derrien *et al*., 2018). True roots of higher plants are effective tissues for nutrient acquisition that have developed root apical meristems and root caps to penetrate soil in an efficient way. Roots have evolved gradually and several times independently in lycophytes and euphyllophytes (Hetherington and Dolan, 2018; Fujinami *et al*., 2020). At the time of transition to land, early vascular plants (e.g. *Zosterophyllum*, *Cooksonia*, *Rhynia*) had rhizoid‐based systems or very rudimentary root axes with limited functionalities and abilities to penetrate the top soil surface (few mm to cm) (Kenrick and Strullu‐Derrien, 2014; Xue *et al*., 2016). The association with microbes and complex microbial communities was therefore essential for plant survival in terrestrial ecosystems (Martin *et al*., 2017; Strullu‐Derrien *et al*., 2018). However, there is currently no fossil‐based evidence for a direct influence of beneficial symbionts on root evolution. The question arises to what extent are alterations in root system architecture launched by plants, under unfavourable conditions, with the aim of generating a habitat for microbes. Such a strategy would enable plants to access their secondary genomes for stress protection and nutrient or water acquisition. An answer might lie in the function and utilisation of hormones.

**Figure 3:**
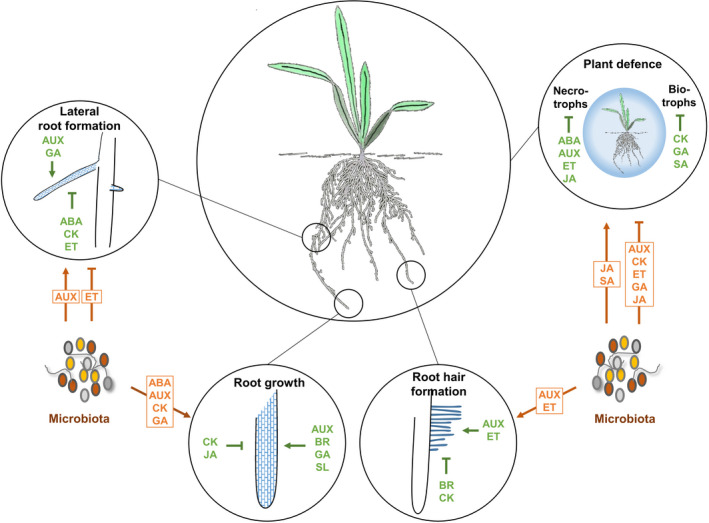
Simplified summary of the functional diversity of plant‐derived and microbial hormone activities on root system architecture and plant defence. Hormone signalling inside plants (in green) activates (↑) or suppresses (↓) lateral root formation, root growth and root hair formation, as well as plant defence against biotrophic and necrotrophic pathogens. Bacterial and fungal microbes are able to produce hormones or modify hormone signalling in plants (in orange), thus altering different aspects of root development and manipulating plant defence. Please note that indicated hormone activities change and can even have opposite effects depending on hormone concentration, hormone homeostasis, plant species, plant developmental stage, environmental stimuli, etc. ABA, abscisic acid; AUX, auxin; BR, brassinosteroid; CK, cytokinin; GA, gibberellic acid; ET, ethylene; JA, jasmonic acid; SA, salicylic acid; SL, strigolactone.

As sessile organisms, plants rely on hormones to orchestrate the different developmental stages, and to add plasticity to plant development under changing environments. Abiotic stress responses, such as drought stress or nutrient deficiencies, require adjustments in root system architecture to penetrate the soil matrix systematically in order to access minerals and water. The primary challenge for plants might be (i) to attract beneficial microbes and (ii) to provide the necessary accommodation infrastructure (habitat). Plant hormones take a central role in the regulation of root system architecture (excellently reviewed in Vanstraelen and Benková, 2012; Petricka *et al*., 2012) (Figure 3). In brief, auxin and CK are among the main regulators of primary root growth through their involvement in cell division and differentiation at the root meristem, respectively. Brassinosteroids (BRs), GA and SL act synergistically to auxin, while JA, ET and ABA support CK‐mediated cell differentiation. Subsequent root cell elongation involves ABA, auxin, CK, ET, JA and SL as inhibiting hormones and GA as an activating hormone. Lateral root development, in turn, is stimulated by auxin, while ABA, CK and ET act antagonistically (Lavenus *et al*., 2013). Auxin, together with ET, is also important for root hair initiation and elongation, which are processes inhibited by BR and CK (Vissenberg *et al*., 2020). The underlying hormone signalling networks are responsive to different environmental stimuli to adjust root system architecture. Nitrogen (N) starvation, for instance, induces primary root growth, whereas enhanced lateral root density and elongation as well as root hair formation help plants to overcome both, low phosphorus (P) and N levels (Li *et al*., 2016; Jia and von Wirén, 2020). Developing horizontal root systems might be especially helpful under stress to recruit beneficial microbes from microbiota‐enriched top‐soil layers. For instance, SLs, which are known to support root colonisation by arbuscular mycorrhizal fungi (AMF), participate in enhanced lateral root formation under P starvation (Lavenus *et al*., 2013). SL activities are likely to represent the initial step to recruit and accommodate AMF, which enhance P supply via their hyphal network upon root colonisation (Parniske, 2008; Li *et al*., 2016). By this means, root symbionts sustain plant fitness by improving nutrient use efficiencies. This further indicates that hormones link the regulation of root development and the establishment of beneficial symbioses.

In terms of plant–microbiome co‐evolution, changes in root system architecture might have been part of plant developmental programmes to generate habitats for beneficial symbionts, a strategy especially important under environmental stress. Various microbes can either produce or otherwise change the levels of phytohormones in the rhizosphere or within a plant and, in doing so, impinge on plant development and stress responses (Hacquard *et al*., 2015; Chanclud and Morel, 2016; Ludwig‐Müller, 2020). Indole acetic acid (IAA), CKs, GAs, ABA and ET have been isolated from microbial culture media (Dodd *et al*., 2010; Spaepen, 2015). Especially in the rhizosphere, hormone‐producing microbes are often non‐pathogenic and even beneficial to plants. Among the well‐known phytohormone‐producing microbes are plant growth‐promoting bacteria (PGPBs) and plant growth‐promoting fungi (PGPFs) (Glick, 2012; Zamioudis and Pieterse, 2012; Bakker *et al*., 2018). Developmental changes caused by microbe‐derived hormones can include alterations in root and shoot growth, as well as in root system architecture and potentially the modification of flowering time (Dodd *et al*., 2010; Spaepen, 2015; Lu *et al*., 2018). Finally, microbes produce hormones that can otherwise benefit a plant, for example, by protecting it against pathogens (hormones as antibiotics or as defence inducers) or by conferring resistance to abiotic stresses (Tsukanova *et al*., 2017; Liu *et al*., 2017a; Rosier *et al*., 2018; Kudoyarova *et al*., 2019). In line with that, root symbionts such as PGPBs affect root cell division and differentiation thereby changing determinants of root system architecture: meristem‐driven indeterminate primary root growth, lateral root and root hair formation (Verbon and Liberman, 2016). PGPB species such as *Pseudomonas simiae* increase lateral root and root hair formation in an auxin‐dependent and JA/ET‐independent manner (Zamioudis *et al*., 2013), while *Pseudomonas* *putida* produces auxin for elongation of primary roots (Patten and Glick, 2002). *Bacillus megaterium* promotes root system architecture via plant CK signalling independently of plant ET and auxin pathways (López‐Bucio *et al*., 2007; Ortíz‐Castro *et al*., 2008). PGPFs such as *Trichoderma* spp. produce ET and/or auxin to increase root hair formation and primary and lateral root growth, respectively (Contreras‐Cornejo *et al*., 2015, 2009). In addition, the ectomycorrhizal fungus *Laccaria bicolor* releases auxin to trigger lateral root development in poplar (*Populus tremula* × *Populus alba*) and Arabidopsis (Felten *et al*., 2009). Those studies exemplify that microbes produce hormones and/or activate plant hormone signalling to alter root system architecture presumably to facilitate their accommodation. This further indicates that changes in root system architecture can originate from sophisticated communication between plants and microbes. It is therefore not surprising that hormones, which were initially thought to exclusively regulate developmental processes, affect interactions with microbes (Robert‐Seilaniantz *et al*., 2011; Pieterse *et al*., 2012; Bürger and Chory, 2019). This might be explained in part by their effects on root development and the provision of habitats for beneficial symbionts.

The fact that microbes can often produce more than one phytohormone, frequently in conjunction with other traits that confer physiological effects to plants or other microbes, makes it difficult to disentangle direct effects by individual hormones. This is probably exemplified by the extreme plant growth‐promoting properties of the bacterium *Pantoea phytobeneficialis* MSR2, which derive from the combined abilities to fix nitrogen, solubilise phosphate, degrade the ET precursor ACC, metabolise JA and produce the plant hormones auxin and CK (Nascimento *et al*., 2020). Altogether, this suit of studies clearly indicates the importance of hormone‐mediated defence signalling and plant developmental processes in shaping the composition of microbiomes.

## PLANT HORMONE EXCRETION AND SIGNALLING CAN SHAPE THE RHIZOSPHERE MICROBIOME

Some functional analogies have been noted between the rhizosphere and the mammalian gut; both constitute the location of nutrient absorption, and contribute to plant/animal health and development. These functions are strongly supported by the organisms’ associated microbiomes (Ramírez‐puebla *et al*., 2013; Hacquard *et al*., 2015). The community composition and identity of microbes in the rhizosphere enhance the plant’s functional capabilities (especially in terms of biotic and abiotic stress resistance) vastly, and it is therefore prudent that plants tightly control the microbial flora therein (Figures 1 and 2) (Turner *et al*., 2013a; Pieterse *et al*., 2016; Bakker *et al*., 2018). The ‘cry‐for‐help’ hypothesis, for example, suggests that plant exposure to pathogens triggers modification of root exudates to signal to the microbial community and promote the accumulation of beneficial microbes in the rhizosphere (Bakker *et al*., 2018; Rolfe *et al*., 2019). This is exemplified by the occurrence of disease suppressive soils after heavy disease outbreaks in the field, which are enriched in microbes with plant‐protective properties. These microbes are able to confer pathogen resistance to subsequent crop generations (Raaijmakers and Mazzola, 2016; Bakker *et al*., 2018; Berendsen *et al*., 2018). In addition, disease‐resistant genotypes often accumulate beneficial microbes in their rhizosphere (Kwak *et al*., 2018; Mendes *et al*., 2018), further suggesting a functional link between plant immunity and microbiome composition. Plant hormones are a key component in the perception of (pathogenic) microbes and subsequent plant immune signalling (for more details of hormone function in plant immunity see Pieterse *et al*., 2012). However, there is an emerging role of plant hormones in shaping root microbiome composition either directly or indirectly, in order to support plant growth under biotic and abiotic stress conditions (Haney and Ausubel, 2015; Carvalhais *et al*., 2017). The capacity for microbes to subvert plant hormone signalling and immune responses is complicating the picture. Effectors secreted by some microbes are capable of targeting specific plant proteins involved in mounting hormone‐dependent immune responses. Readers interested in this subject are referred to (Nobori *et al*., 2018; Han and Kahmann, 2019). Specific plant hormones can be released into the rhizosphere and may have a direct impact on plant‐interacting microbes and the root associated microbiome at large (Xu *et al*., 2018b; Carvalhais *et al*., 2019; Nasir *et al*., 2019; Liu *et al*., 2020). Evidence exists for the presence of most plant‐derived hormones in root exudates (Torrey, 1976; Reddy *et al*., 1989; Faure *et al*., 2009). On the other hand, there are potential indirect influences hormones have on shaping root exudates used to communicate with the microbial community (Schreiner *et al*., 2011; Carvalhais *et al*., 2015). The direct versus indirect effect of plant hormones on shaping the microbiome is not always clear and should be taken into careful consideration when designing and interpreting studies. The quantitative aspect of hormones and their interaction with microbes is also important, particularly when considering hormones released into the rhizosphere, and often not well represented in microbiome research. Quantification of plant hormones is challenging, with hormones present at pg g^−1^ or ng g^−1^ concentrations in fresh plant tissue (Pan *et al*., 2010). Methods for more accurate quantification are however improving (Fu *et al*., 2011; Wang *et al*., 2020).

### Direct and indirect effects of plant hormones on microbial communities

#### Strigolactones – more than facilitators of specific plant symbioses

SLs are a group of compounds relatively recently recognised as plant hormones with functions in shoot branching, root system architecture, parasitic weed germination and plant–microbe communication (Yoneyama *et al*., 2008; Al‐Babili and Bouwmeester, 2015; Clear and Hom, 2019; Aliche *et al*., 2020). They also display complex cross‐talk with other hormones and are therefore involved in many aspects of plant growth and development (Cheng *et al*., 2013; Omoarelojie *et al*., 2019). SLs have a role in classical plant symbioses with AMF and nodulating rhizobia (Steinkellner *et al*., 2007; Foo and Davies, 2011; Clear and Hom, 2019). Although an SL receptor has not been characterised in AMF, so far, treatment with SL induces a variety of fungal responses, including morphological and transcriptional changes, and the stimulation of the release of secreted proteins, which support plant colonisation (Lanfranco *et al*., 2018). SLs are long known to induce hyphal branching of AMF, a process initiated before colonisation of plant roots (Akiyama *et al*., 2005, 2010; Akiyama and Hayashi, 2006; Yoneyama *et al*., 2008). The hyphal branching phenotype is generally not observed in other soil‐borne fungi (Steinkellner *et al*., 2007). Higher concentrations of SLs (>10 μm) have, however, been shown to reduce hyphal branching with some phytopathogenic fungi (Dor *et al*., 2011), reduce the growth rate of a beneficial fungus (*Mucor* sp.) and also be required for a *Mucor* sp. to promote plant growth (Rozpadek *et al*., 2018).

Soybean (*Glycine max*) and *Lotus japonicus* mutants deficient in SL synthesis display reduced nodule numbers and this phenotype can be rescued by exogenous SL application (10 μm) (Foo and Davies, 2011; Rehman *et al*., 2018). Additionally, rhizobial infection has been shown to alter the expression of SL biosynthesis genes (Rehman *et al*., 2018). Further, SLs have been implicated in increased swarming and motility of rhizobia (Tambalo *et al*., 2014; Peláez‐Vico *et al*., 2016). In light of the importance of SLs in plant–microbe symbiosis formation, several studies have used community profiling, by amplicon sequencing, to address the impact of SLs on the wider bacterial and fungal microbiomes (Carvalhais *et al*., 2019; Nasir *et al*., 2019; Liu *et al*., 2020). Studies suggest that SL signalling is involved in shaping fungal and bacterial rhizosphere communities. Several specific fungal, but not bacterial taxa were differentially abundant in the Arabidopsis SL biosynthesis mutant *more axillary growth 4* (*max4*). The abundance of biocontrol *Penicillum* and *Epicoccum* sp. and the *Plectosphaerella cucumerina* pathogen was reduced, while that of species from two other pathogen groups (*Hypocreales* and *Ramularia*) was increased in the mutant rhizosphere (Carvalhais *et al*., 2019). In rhizospheres of soybean plants overexpressing SL biosynthesis and signalling genes, representatives of *Fusarium solani*, *Rhizobiaceae*, predatory *Bdellovibrio* and *Shinella* were more abundant (Liu *et al*., 2020). Conversely, SL biosynthesis‐ and signalling‐deficient rice (*Oryza sativa*) mutants showed a reduction in several beneficial groups of bacteria (plus *Bdellovibrio*) in the mutant rhizospheres but also a decrease in the pathogenic fungus *Olpidium brassicae* (Nasir *et al*., 2019). Taken together, SL clearly affects the shape of both fungal and bacterial rhizosphere communities but without an obvious positive or negative influence.

#### Abscisic acid and auxin – carbon source for microbes and stimulator of microbial signalling

ABA is canonically involved in abiotic stress tolerance and seed dormancy, the antagonistic relationship with GA breaking dormancy in favourable conditions (reviewed in Verma *et al*., 2016). Auxins, such as IAA, are implicated in plant growth and development but more recently their involvement in stress tolerance has become apparent (Shani *et al*., 2017). ABA also supports plant–AMF interactions (Herrera‐Medina *et al*., 2007; Martín‐Rodríguez *et al*., 2011; reviewed by Stec *et al*., 2016) and shows a positive correlation with the abundance of SL. ABA affects nodulation (Suzuki *et al*., 2004), likely by inhibition of CK biosynthesis (Ding *et al*., 2008), which is vital for nodule organogenesis. ABA and IAA can be used as the sole carbon source by some rhizobacteria, for example, a *Rhodococcus* sp. and a *Novoshingobium* sp. can use ABA (Belimov *et al*., 2014), while *P. putida* strain 1290 can feed on IAA (Leveau and Lindow, 2005). In these studies the concentrations of ABA and IAA are far greater than those previously reported to be present in plant tissues (Belimov *et al*., 2014; Rehman *et al*., 2018); however, no attempt was made to establish a lower limit. Secretion of ABA or IAA into the rhizosphere could be a mechanism for plants to select microbes capable of using these as a carbon source. Exogenous ABA application has been shown to induce vast gene expression changes in the endophyte *Aspergillus nidulans* (Xu *et al*., 2018a), and IAA induced production of invasive filaments in *Saccharomyces cerevisiae* (Prusty *et al*., 2004), providing evidence for the perception of ABA by microbes and subsequent changes in growth processes. Most interestingly, IAA application increased the antimicrobial activity of several strains of the actinobacterial Streptomycetaceae family isolated from Arabidopsis roots towards *Escherichia coli* and *Bacillus subtilis* (van der Meij *et al*., 2018). Actinobacteria are one of the most dominant phyla in the plant root microbiome and are known for their antimicrobial compound‐producing capabilities.

#### Jasmonic acid, salicylic acid and ethylene – defence hormones with wider effects on microbial communities

SA is a well‐studied defence hormone and perceived by SA immune signal receptors NPR1, NPR3 and NPR4. The latter two act in conjunction with NPR1 to regulate different SA‐mediated immune responses (Dong, 2004; Ding *et al*., 2018; Li *et al*., 2019). Furthermore, SA and NRP1 are known to interact with JA signalling pathways to adjust immune signalling in response to pathogen attack (Li *et al*., 2004; Spoel *et al*., 2007; Leon‐Reyes *et al*., 2010). Mutants deficient in NPR1 function have reduced endosphere alpha diversity, and can restrict colonisation by endophytes (Hein *et al*., 2008; Chen *et al*., 2020a). These mutants maintain their ability to synthesise SA and in fact may have elevated levels of SA under some conditions (Rayapuram and Baldwin, 2007). The SA‐associated alpha diversity reduction that was observed in root endospheres extended into the rhizosphere (Hein *et al*., 2008; Doornbos *et al*., 2011) while constitutive degradation of SA reduced alpha diversity in the endosphere but not rhizosphere of tomato or Arabidopsis (Doornbos *et al*., 2011; French *et al*., 2019). Moreover, direct SA application affected microbes in bulk soil indicating SA plant signalling‐independent effects on microbial communities (Lebeis *et al*., 2015). Collectively, these results suggest that SA may act via canonical signalling pathways, via interaction with other hormones such as JA or directly on community members to promote or inhibit their growth.

Exogenous JA application, used to activate JA signalling, has been shown to increase Arabidopsis rhizosphere alpha diversity along with an enrichment of several potentially beneficial microbial taxa (Carvalhais *et al*., 2013), whilst Arabidopsis mutants deficient in JA conversion to its bioactive form (JA‐isoleucine [JA‐Ile]) show a reduced diversity (Doornbos *et al*., 2011). Results are not always consistent across plant species and tissues. A contrasting role of JA in epiphytic Arabidopsis leaf communities and wheat (*Triticum aestivum*) root endosphere community composition has been reported (Kniskern *et al*., 2007; Liu *et al*., 2017b). Again, however, actinobacteria were implicated as the major component of community changes. JA‐mediated microbiome assembly is likely realised, indirectly, via root exudate composition changes. Mutants deficient in JA signalling display reductions, in their root exudate profiles, of several positive bacterial chemotaxis compounds and compounds mediating inter‐bacterial interactions (Carvalhais *et al*., 2015).

Such changes in actinobacteria abundance were also observed to be affected by ET, which often acts synergistically with JA in defence signalling. However, ET activity appears not to be restricted to the root endosphere but can also alter additional plant–microbiome processes (Ravanbakhsh *et al*., 2018). When grown in an intercropping system, cyanide released by cassava (*Manihot esculenta*) roots was perceived by neighbouring peanut (*Arachis hypogaea*) roots to trigger ET excretion. This exogenous ET resulted in an increase in rhizosphere alpha diversity, especially again the abundance of actinobacteria, whilst the abundance of acidobacteria was reduced (Chen *et al*., 2020b). In addition to reassembling the rhizosphere communities, those actinobacteria improved nutrient supply and seed production in peanuts.

Taken together, classical defence hormones have functions in microbiome assembly that go beyond the endosphere and intrinsic plant defence signalling. It highlights how microbiome studies enable us to assign previously unknown functions to defence hormones such as direct effects or plant‐independent effects on microbe fitness.

#### Cytokinin, gibberellic acid and brassinosteroids – known developmental regulators that steer plant–microbe interactions

Plant hormones such as CK, GA and BR are underrepresented in microbiome research. Many studies, however, consider the influence these hormones have on specific beneficial and pathogenic plant–microbe interactions (Nakashita *et al*., 2003; Choi *et al*., 2010; Jiang *et al*., 2013; Reusche *et al*., 2013; Yu *et al*., 2018), with potentially broader implications in shaping plant microbiomes.

CKs are known to be key for the formation of nodules in legumes. Exogenous CK induces the formation of pseudonodules in non‐rhizobia‐infected nodulating *L. japonicus* (Heckmann *et al*., 2011) and several other nodulating legumes, but not in non‐nodulating legumes or non‐legumes (Gauthier‐Coles *et al*., 2019). This effect can be blocked by ET (Heckmann *et al*., 2011). The enhancement of pathogen resistance promoted by CK has been shown in several plant–pathogen systems, which seems to be synergistic with, and dependent on, SA‐responsive pathways (Choi *et al*., 2010; Jiang *et al*., 2013; Reusche *et al*., 2013). Contrastingly, CK appears to support the progression of fungal biotrophic pathogens and stimulate beneficial AMF interactions in pea (*Pisum sativum*) (Walters and McRoberts, 2006; Chanclud *et al*., 2016; Morrison *et al*., 2017; Goh *et al*., 2019).

GA and BR are involved in nodulation and AMF formation (excellently reviewed by McGuiness *et al*., 2019). While GA has a rather suppressive effect on arbuscule formation, BRs seem to support it (Foo *et al*., 2016, 2013). The impact of these two hormones on nodulating rhizobia is far less clear – with promotion or inhibition being dependent on concentration and/or species – but it has been speculated that cross‐talk with ET is, in part, responsible (McGuiness *et al*., 2019). Outside of classical mycorrhizal and rhizobial symbioses, BRs have been shown to promote disease resistance of plants to a broad range of plant pathogens (Nakashita *et al*., 2003). Earlier studies considered the influence of spray treatment of several species of flowering plants with varying concentrations of GA or IAA on fungal load, assessed by weighing culturable fungi isolated from soils (Sullia, 1968; Gupta, 1971). The response of soil fungi was extremely plant species‐ and hormone concentration‐dependent. Often an intermediate hormone concentration provided an increase in fungal load and higher concentrations reduced the load back to basal levels. In these two studies hormones were applied as foliar sprays, indicating that some signal must be systemically propagated from shoots to roots to impact soil fungi and promote their growth, possibly via root exudates. In conclusion, given the importance of these hormones in interactions with both beneficial and pathogenic microbes, future studies using NGS techniques (e.g. metatranscriptomics with microbiome profiling and plant RNA sequencing) should help to assess their impact on the wider microbiome.

### Hormone‐dependent changes in root exudates

There is a huge array of research considering the effect of specific root exudates on root‐associated microbiomes (Chaparro *et al*., 2013; Stringlis *et al*., 2018; Huang *et al*., 2019; Voges *et al*., 2019). Readers more widely interested in this topic are referred to some excellent reviews (Dennis *et al*., 2010; Doornbos *et al*., 2012; van Dam and Bouwmeester, 2016; Sasse *et al*., 2018; Guerrieri *et al*., 2019; Preece and Peñuelas, 2020), while we focus here only on hormone effects on exudation. Root exudates can impact microbial communities and selectively modify them by changing the spatial organisation, gene expression or abundance of particular taxa. In addition to serving as carbon sources, exudates can have antimicrobial activity, or they can act as signalling molecules. These are likely indirectly affected by plant hormone signalling, and examples exist of the impact hormones have on root exudates (Schreiner *et al*., 2011; Carvalhais *et al*., 2015).

Specific root exudates can selectively modify the microbiome by exploiting the substrate preferences of different bacterial species (Badri *et al*., 2013; Zhalnina *et al*., 2018). Badri *et al*. (2013) showed that certain compounds from Arabidopsis root exudates promote the growth of groups of operational taxonomic units (OTUs), which are clusters of sequences, identified from amplicon sequencing data, and considered as representative of the same species. Some compounds are able to differentially promote and inhibit different OTUs. Phenolic compounds were found to be of particular importance, as they are able to modify the growth of more OTUs than other compounds. Caffeic acid and phenolic compounds have also been implicated as exudates that can aid in disease resistance (Ling *et al*., 2013; Gu *et al*., 2016). Caffeic acid and chlorogenic acid in approximately the 10–100 μm range inhibited the growth of a fungal pathogen (Ling *et al*., 2013). Moreover, SA was a major component of root exudates and posited as involved in the repression of fungal growth (Wu *et al*., 2009; Ling *et al*., 2013).

JA or SA application has been shown to induce higher abundance of glucosinolates in exudates of *Brassica rapa* (Schreiner *et al*., 2011), a secondary metabolite with antimicrobial activity against *Brassica napus* to *Plasmodiophora brassicae* (Xu *et al*., 2018b). Although not specifically in root exudates, lower aliphatic glucosinolate levels (and biosynthesis gene expression levels thereof) have also been detected in Arabidopsis IAA perception mutants, after drought stress induction (Salehin *et al*., 2019). This implicates a function of auxins, not only JA and SA, in the production of glucosinolates and illustrates again the complexity and abundance of cross‐talk between hormone signalling pathways and their regulation of metabolite production. Arabidopsis mutants deficient in JA signalling showed changes in their root exudate profiles, which correlated with shifts in microbiome composition. In addition to a lower abundance of several compounds known to act as chemotactic signals, kaempferol with its known antimicrobial activity was less abundant in mutant lines. In addition, the abundance of several specific compounds including sugars and amino acids was found to correlate significantly with the abundance of specific OTUs assigned to genera such as *Bacillus* and *Pseudomonas*, or to the *Clostridiales* order (Carvalhais *et al*., 2015).

Benzoxazinoids (BXs) are a group of compounds present in many cereal crops (such as maize [*Zea mays*], rye [*Secale cereale*] and wheat) and known for their role in plant defence (Hu *et al*., 2018). JA and ET application has been implicated in the production of BX in maize (Dafoe *et al*., 2011). Several studies have shown the impact of BXs in fungal and bacterial microbial communities in the maize rhizosphere. The overall effect on pathogen abundance is however ambiguous. While some studies report decreases in pathogenic microbes (Kudjordjie *et al*., 2019; Cotton *et al*., 2019), others report increases (Cadot *et al*., 2020). The BX‐deficient phenotype (herbivory susceptibility, reduced JA and reduced SA) has been shown to be mediated by the BX breakdown product 6‐methoxy‐benzoxazolin‐2‐one (MBOA) and is dependent on microbiome changes (Hu *et al*., 2018). These changes could be caused directly by MBOA in exudates or by signalling to condition soils with a broader range of metabolites (Cotton *et al*., 2019).

Taken together, belonging to the class of low‐molecular weight compounds, plant hormones and secondary metabolites are highly mobile and can affect processes by their direct biological activity (Erb and Kliebenstein, 2020). Importantly, hormones can mediate their function through metabolite synthesis and excretion and we are just beginning to understand the impact of this mechanism on microbiome assembly and plant fitness (Leach *et al*., 2017; Nobori *et al*., 2018; Huang *et al*., 2019).

## MICROBES PRODUCE PHYTOHORMONES AND MANIPULATE HORMONE SIGNALLING TO INTERACT WITH PLANTS AND OTHER MICROBES

Plant‐associated microbes produce a broad range of hormones and hormone‐like substances, or possess enzyme activities, which alter hormone levels in the plant endosphere, phyllosphere and rhizosphere (Dodd *et al*., 2010; Spaepen, 2015). Some of these microbe‐derived hormones have obvious effects on plant physiology or support host colonisation, for example, by interfering with plant defence responses (Figure 3). Other microbial hormones can serve as a nutrient source or have antimicrobial activities, and may thus influence neighbouring microbial communities directly. However, these direct effects on microbes may be locally restricted and confined to specific niches, making them hard to trace. In addition, such effects might be masked by plant‐derived hormones. Hence, available information on broader impacts of microbe‐derived hormones on soil‐resident or plant‐associated microbiomes is currently rather limited.

### Auxin – growth hormone widely produced by bacterial and fungal microbes

Auxin and CK affect plant growth, which can either be beneficial, if they support general plant growth especially under unfavourable conditions, or detrimental, if they are being exploited for feeding a pathogen (Boivin *et al*., 2016). Several pathogenic and non‐pathogenic bacterial and fungal microorganisms are capable of auxin (mostly IAA) biosynthesis. The existence of biosynthesis pathways for auxin has been proposed based on the detection of IAA and metabolic intermediates in culture media and the presence of IAA biosynthesis genes in bacterial and fungal genomes. In most of these pathways, the aromatic amino acid tryptophan serves as precursor (Spaepen *et al*., 2007; Spaepen and Vanderleyden, 2011; Patten *et al*., 2013; Duca *et al*., 2014; Chanclud and Morel, 2016; Kunkel and Harper, 2018; Han and Kahmann, 2019; Meents *et al*., 2019; Morffy and Strader, 2020). Microbe‐derived auxin may serve several functions especially in interaction with plants. Some evidence suggests that in microbes, auxin has a physiological role and serves as signalling molecule (Spaepen *et al*., 2007; Patten *et al*., 2013). The IAA precursor tryptophan, IAA itself and other auxins have been shown to induce bacterial IAA biosynthesis genes. In a similar way, plant‐derived metabolites such as flavonoids can induce IAA biosynthesis, for example, in rhizobia (Spaepen *et al*., 2007; Spaepen and Vanderleyden, 2011; Patten *et al*., 2013). Microbial IAA production and secretion may aid in regulation of pH homeostasis. In bacteria, IAA may serve as signalling molecule in biofilm formation, population growth and behaviour especially under unfavourable conditions such as nutrient limitation, temperature changes or acidic pH (Spaepen *et al*., 2007; Spaepen and Vanderleyden, 2011; Patten *et al*., 2013; Duca *et al*., 2014; Kunkel and Harper, 2018). Antimicrobial activity has been attributed to the weak acid IAA, and some bacteria may perceive IAA as a signal to induce their antibiotic production, possibly in order to increase the ability to compete with other microbes for limited resources (Duca *et al*., 2014). Some bacteria can actively degrade IAA and may use it as a carbon and nitrogen source. IAA degradation may also serve as a means to protect bacteria from the antimicrobial activity of IAA, and has been observed in conjunction with chemotaxis towards IAA in *P. putida* (Scott *et al*., 2013; Duca *et al*., 2014). Among competing microorganisms in a complex chemical environment such as the rhizosphere, this trait may thus help to open up ecological niches. On the other hand, the growth‐promoting properties, for example, of the PGPB *Burkholderia phytofirmans* depend upon its abilities to degrade auxin, especially in an environment with high IAA levels (Zúñiga *et al*., 2013). High levels of IAA produced by a bacterial community can affect Arabidopsis root growth. Interestingly, strains of auxin‐degrading bacteria can interfere with this negative chemical signalling and restore root growth (Leveau and Lindow, 2005; Finkel *et al*., 2020).

Auxin can contribute to pathogenesis and virulence of gall‐forming and other plant pathogenic bacteria and fungi (Spaepen *et al*., 2007; Kunkel and Harper, 2018; Han and Kahmann, 2019). The tumour‐forming soil bacterium *Agrobacterium tumefaciens* can change plant hormone biosynthesis and activate cell proliferation directly by integrating genes for auxin (and CK) biosynthesis into the host genome, resulting in the formation of the typical crown galls (Gohlke and Deeken, 2014). Other gall‐forming plant pathogens, such as *Pseudomonas savastanoi* pv. *savastanoi*, which causes knot disease in olive (*Olea europaea*), are able to produce and secrete these hormones for tumour formation on their own (Dodueva *et al*., 2020). Fungi which cause tumours or other plant organ deformations (e.g. *Ustilago maydis*, *Taphrina* spp.) possess auxin biosynthesis genes, but the function of these genes in infection and disease symptom development is not entirely clear (Tsai *et al*., 2014; Ludwig‐Müller, 2015; Chanclud and Morel, 2016; Han and Kahmann, 2019; Dodueva *et al*., 2020). The hemibiotrophic rice blast fungus *Magnaporthe oryzae* and some *Colletotrichum* spp., as well as the biotrophic rust fungus *Puccinia graminis* f. sp. *tritici*, produce IAA during their biotrophic growth phase (Ludwig‐Müller, 2015; Chanclud and Morel, 2016). In addition, local accumulation of IAA in plants either through microbial secretion or after microbe‐induced elevation of plant IAA levels supports plant colonisation, most likely through the suppression of (SA‐mediated) host defence responses. Through the induction of expansins, local accumulation of auxin can also contribute to cell wall loosening and thus provides a microbial means to create host entry sites or induce cellular hypertrophy to feed the invader (Spaepen *et al*., 2007; Kazan and Manners, 2009; Patten *et al*., 2013; Duca *et al*., 2014; Kunkel and Harper, 2018; McClerklin *et al*., 2018).

As shown for PGPBs, microbial auxin contributes to changes in plant physiology such as enhanced root growth and root hair formation and altered root system architecture (Figure 3). The resulting surface area extension may lead to the provision of more root exudates, which could serve as growth substrate for microbial communities in the rhizosphere (Barea *et al*., 2005; Spaepen *et al*., 2007; Dodd *et al*., 2010; Vacheron *et al*., 2013; Cassán *et al*., 2014; Duca *et al*., 2014; Spaepen, 2015; Kudoyarova *et al*., 2019). The capability of PGPBs to maintain plant growth even under nutrient deficiency or other abiotic stress conditions has been attributed to the microbes’ ability to alter root development through the production of auxin (Marulanda *et al*., 2009; Belimov *et al*., 2015; Rolli *et al*., 2015; Zhou *et al*., 2016; Kudoyarova *et al*., 2019). Genetically modified strains of *Azospirillum brasilense*, in which IAA biosynthesis was enhanced, increased shoot biomass in wheat seedlings. Interestingly, the IAA overproducing strains had a significant impact on rhizosphere microbiota, whereby the effects on rhizobacteria and fungi were different, depending on the promoter (constitutive versus root exudate‐responsive) by which bacterial IAA biosynthesis was driven. Whether the observed effects of bacterial IAA on the microbial communities was direct or indirect via plant‐mediated effects needs to be determined (Baudoin *et al*., 2010).

Changes in Arabidopsis root system architecture also accompany colonisation by the beneficial, plant growth‐promoting root endophyte *Serendipita indica* (formerly *Piriformospora indica*) (Sirrenberg *et al*., 2007). *Serendipita indica* can produce IAA in culture media at levels that may be sufficient to impact on primary and especially lateral root formation (Sirrenberg *et al*., 2007; Meents *et al*., 2019). In interaction with barley (*Hordeum vulgare*), *S. indica*‐derived IAA was shown to be required for root colonisation during biotrophic growth, but not for plant growth promotion (Hilbert *et al*., 2012). It has been shown recently that other fungus‐derived metabolites can induce local auxin responses and may thus be responsible for initiation of lateral root formation (Inaji *et al*., 2020). Other beneficial plant symbionts such as many *Rhizobium* spp. can secrete IAA, and it has been assumed that they use it together with altering plant auxin transport to locally change auxin homeostasis during root nodule formation in legumes (Spaepen and Vanderleyden, 2011; Boivin *et al*., 2016; Mathesius, 2020). In a similar way, ectomycorrhizal fungi can produce auxin, which seems to support fungal colonisation, and can alter host root morphology (Boivin *et al*., 2016; Chanclud and Morel, 2016). In contrast, although IAA also promotes fungal invasion and AM formation especially at early stages, AMF seem to be unable to synthesise auxin (Das and Gutjahr, 2020; Ludwig‐Müller, 2020).

### Cytokinins – used by microbes as antimicrobial agents and for host colonisation

Similar to auxin, CKs regulate cell division and differentiation, and have a major effect on plant growth processes (Figure 3) (Werner and Schmülling, 2009; Cammarata *et al*., 2019). It is thus not surprising that microbe‐associated IAA biosynthesis is often accompanied by the ability to produce CKs (Morris, 1986; Costacurta and Vanderleyden, 1995; Jameson, 2000; Boivin *et al*., 2016; Kudoyarova *et al*., 2019). Adenine molecules serve as backbones in microbial CK biosynthesis. Like auxin, CKs produced directly or indirectly by bacterial pathogens such as *A. tumefaciens* or *P. savastanoi* contribute to gall or tumour formation (Costacurta and Vanderleyden, 1995; Jameson, 2000; Kazan and Lyons, 2014; Hinsch *et al*., 2015; Spaepen, 2015; Sørensen *et al*., 2018; Spallek *et al*., 2018). The presence of CK biosynthesis genes and the production of CKs or CK‐like molecules have also been shown for fungal pathogens, for example, for *Claviceps purpurea*, *U. maydis*, *Leptosphaeria maculans*, *M. oryzae*, and *Fusarium pseudograminearum* (Bruce *et al*., 2011; Hinsch *et al*., 2015; Chanclud *et al*., 2016; Trdá *et al*., 2017; Sørensen *et al*., 2018). In addition, bacterial and fungal genomes harbour histidine kinases with similarities to plant ET or CK receptors (Hérivaux *et al*., 2017; Kabbara *et al*., 2018). The histidine kinase PcrK of the plant pathogenic bacterium *X. campestris* pv. *campestris* can sense a plant CK, and, upon perception, supports bacterial growth under oxidative stress. PcrK orthologs are conserved in other plant‐associated bacterial genera, for example, *Pseudomonas* or *Dickeya* (Wang *et al*., 2017) and it has been proposed that PcrKs act as a bacterial virulence factor by providing a means to cope with plant defence‐related oxidative stress. Consistent with this, analysis of microbial mutants revealed that fungal‐derived CKs often have virulence functions and likely suppress host defence responses (Spallek *et al*., 2018; Han and Kahmann, 2019). CKs are often found in (hemi)biotrophic fungi and have been linked to the formation of so called ‘green islands’, that is, areas around fungal infection sites, where senescence processes are thought to be inhibited and nutrient fluxes redirected in order to support the biotrophic growth phase of the intruder (Walters and McRoberts, 2006; Walters *et al*., 2008; Spallek *et al*., 2018). However, CK production is not restricted to pathogenic fungi, and may support some general physiological processes, for example, during hyphal growth, nutrient uptake, growth under unfavourable conditions and sexual reproduction (Chanclud and Morel, 2016). CKs accumulate in AMF‐colonised plants, but it is unclear if they derive from the fungus or the host plant, and how much they contribute to the infection process (Boivin *et al*., 2016; Chanclud and Morel, 2016; Bedini *et al*., 2018; Das and Gutjahr, 2020). CK production has been assigned to some ectomycorrhizal fungi, but whether there is a functional role in establishing the symbiosis is not known (Morrison *et al*., 2015; Boivin *et al*., 2016). Rhizobia can produce CKs alongside IAA, and, through the release of Nod factors, induce endogenous CK accumulation and nodule formation. However, bacteria‐produced CKs alone seem not to be sufficient for nodule formation (Frugier *et al*., 2008; Kisiala *et al*., 2013; Boivin *et al*., 2016; Miri *et al*., 2016; Foo, 2020).

The contribution of CK biosynthesis of PGPBs to alter plant development is less well documented, and probably often masked by the effects of concomitantly produced IAA or GAs (Ortíz‐Castro *et al*., 2009; Sgroy *et al*., 2009; Dodd *et al*., 2010; Vacheron *et al*., 2013; Spaepen, 2015; Kudoyarova *et al*., 2019). At least at high levels, exogenous application of CKs may even inhibit root growth, potentially through the induction of ET production, and in some cases plant growth inhibition has been connected to bacterial CK biosynthesis (Dodd *et al*., 2010). Bacterial CK can act as biocontrol agent against pathogens: When applied to plant leaves, *Pseudomonas fluorescens* G20‐18 can activate plant resistance to pathogenic *Pseudomonas syringae*, and this is dependent on the bacterium’s ability to produce CK and the plant’s ability to perceive it (Großkinsky *et al*., 2016; Akhtar *et al*., 2020). Microbial production of CKs may also improve abiotic stress resistance, for example, against drought (Xu *et al*., 2012; Liu *et al*., 2013; Kudoyarova *et al*., 2019). Moreover, it has been reported that microbial‐produced CK can increase the release of root exudates, for example, amino acids, into the rhizosphere, and may thus have a broader impact on the rhizosphere microbiome (Kudoyarova *et al*., 2014).

### Ethylene – a balancing act between microbial production and degradation

The inhibitory effects of high levels of (microbe‐produced) auxin or CK on root growth are often linked to simultaneously elevated levels of the gaseous hormone ET (Dodd *et al*., 2010; Glick, 2014; Gamalero and Glick, 2015). ET can inhibit the cell cycle and negatively affect cell division and meristem size in leaves and roots. ET inhibits root elongation and lateral root formation, but can increase the number of root hairs (Figure 3) (Dodd *et al*., 2010; Street *et al*., 2015; Van de Poel *et al*., 2015). Plants produce ET in response to a variety of stresses including pathogen attack, high salinity, flooding, heat, drought, nutrient deficiency and heavy metal toxicity, and can impair leaf and root growth as well as yield (Glick, 2012, 2014; Dubois *et al*., 2018). In plants, ET is synthesised from the amino acid methionine through sequential conversion into *S*‐adenosyl methionine and then into ACC through ACC synthase. ET production can be adjusted through the regulation of ACC synthase abundance/activity or the availability of the ET precursor ACC (Dubois *et al*., 2018; Nascimento *et al*., 2018). ET can have positive or negative effects on fungal spore germination and hyphal growth, and can restrict root colonisation by specific endophytes or symbionts (Tudzynski and Sharon, 2002; Zhu *et al*., 2017; Liu *et al*., 2017a). Exogenous application of ET restricts plant colonisation by mycorrhizal fungi and limits arbuscule formation. At various stages, ET can also interfere with the formation and function of nodules (Guinel, 2015; Bedini *et al*., 2018; Das and Gutjahr, 2020; Foo, 2020; Ludwig‐Müller, 2020; Mathesius, 2020). Therefore, rhizobia have developed strategies to manipulate the plant ET biosynthesis pathway likely in order to reduce ET levels within the root. Several rhizobial species contain ACC deaminase enzymes, which can catabolise exuded ACC into ammonia and α‐ketobutyrate. In this way, rhizobia may be able to reduce the local amount of the ET precursor ACC, thus limiting its negative effect on nodule formation and/or function (Ma *et al*., 2002; Ma *et al*., 2003; Gamalero and Glick, 2015). In fact, overexpression of ACC deaminase in *Mesorhizobium loti* led to an increase in the number of nodules formed in *L. japonicus* (Conforte *et al*., 2010). In a similar way, co‐infection of *Bradyrhizobium japonicum* with ACC deaminase‐expressing rhizobacteria improved nodulation in mung bean (*Vigna radiata*) (Shaharoona *et al*., 2006). ACC deaminase‐producing bacteria also supported colonisation and arbuscule formation by the AMF *Gigaspora rosea* in cucumber (*Cucumis sativus*) (Gamalero *et al*., 2008). Especially under stress conditions, for example, high salinity, plants produce high amounts of ACC and ET. If not converted into ET, ACC can be excreted from roots into the rhizosphere (Glick, 2014; Nascimento *et al*., 2018; Liu *et al*., 2019). High ACC deaminase activity is a frequent feature of free‐living or endophytic PGPBs and fungi (Ma *et al*., 2003; Glick, 2005; Dodd *et al*., 2010; Orozco‐Mosqueda *et al*., 2020). ACC deaminase‐producing bacteria are more abundant in the rhizosphere of plants grown under stress conditions (Nascimento *et al*., 2018). PGPBs may use their ACC deaminase activity to convert ACC and utilise it as a growth substrate outside the plant. It has been proposed that by using up ACC outside plant roots, PGPBs may create a sink that can protect plants from growth‐inhibiting levels of stress‐related ET and alleviate detrimental effects of unfavourable environmental conditions (Glick, 2005; Glick, 2014; Gamalero and Glick, 2015; Nascimento *et al*., 2018; Kudoyarova *et al*., 2019; Orozco‐Mosqueda *et al*., 2020). In PGPBs, the ability to produce IAA is often found together with ACC deaminase activity. Reducing growth‐inhibiting levels of IAA‐induced ET may help to increase the plant growth‐promoting potential of bacterial IAA in the presence and absence of plant stress (Glick, 2014; Belimov *et al*., 2015; Kudoyarova *et al*., 2019; Orozco‐Mosqueda *et al*., 2020). Whether ACC consumption by microbes affects rhizosphere microbiomes directly is not known.

Some pathogenic and non‐pathogenic bacterial and fungal microorganisms can produce ET, often using methionine as a precursor. *Botrytis cinerea*, *Fusarium oxysporum* f. sp. *tulipae*, *Alternaria alternata*, *Ralstonia solanacearum* and *P. syringae* pathovars are among the ET‐producing plant pathogens (Arshad and Frankenberger, 1990; Nagahama *et al*., 1992, 1994; Weingart and Völksch, 1997; Weingart *et al*., 2001; Tudzynski and Sharon, 2002; Valls *et al*., 2006; Kazan and Lyons, 2014; Zhu *et al*., 2017). In some *Colletotrichum* spp., which cause post‐harvesting diseases on fruits, (fruit‐derived) ET supports spore germination, hyphal growth and appressorium formation, thus supporting fungal virulence (Flaishman and Kolattukudy, 1994). The brown spot pathogen *Cochliobolus miyabeanus* produces ET to promote infection of rice plants, most likely through the suppression of cellular defence responses (Van Bockhaven *et al*., 2015). Some pathovars of *P. syringae* can produce ET in culture and during infection *in planta*. However, disruption of the bacterial gene encoding the ET‐forming enzyme reduced pathogen virulence in one pathovar, but not in another, suggesting that pathogen‐derived ET is not a general virulence factor in pathogenic plant–*P. syringae* interactions (Weingart and Völksch, 1997; Weingart *et al*., 2001). Despite the negative effect ET has on nodule formation, some rhizobia, for example, *B. japonicum*, can produce ET in culture media supplemented with methionine (Boiero *et al*., 2007). ET production has been shown for some ectomycorrhizal fungi, and the hormone seems to support symbiosis, potentially through the interference with host immunity or by inducing plant auxin production and lateral root formation (Splivallo *et al*., 2009; Boivin *et al*., 2016; Chanclud and Morel, 2016). Organic compounds such as carbohydrates, amino acids (especially methionine) and organic acids present in root exudates can stimulate microbial ET production. Consequently, the rhizosphere can be rich in ET‐producing microbes (Arshad and Frankenberger, 1990, 1991). ET biosynthesis has been shown for a broad range of rhizobacteria including *Azospirillum*, *Azotobacter* and *Bacillus* spp. (Nagahama *et al*., 1992; Cassán *et al*., 2014). Due to its potentially suppressive effect on other soil microbes, microbe‐produced ET may influence soil microbial populations directly (Smith, 1973; Smith and Cook, 1974) and rhizosphere ET levels can reach concentrations that can affect plant development (Arshad and Frankenberger, 1991). Although generally considered as root growth inhibitory, microbial ET production may enhance root hair formation/elongation (Figure 3) (Ribaudo *et al*., 2006; Galland *et al*., 2012; Vacheron *et al*., 2013).

Together, the combined potential of soil microbes to elevate or reduce ET levels in and around plants may provide a greater phenotypic plasticity, especially in response to various stress factors (Ravanbakhsh *et al*., 2018). Given the potential antimicrobial activity of ET and the usability of its precursor ACC as nutrient source, microbial activities that change local concentrations of ET or ACC may impact on co‐habitation in microbial niches in the rhizosphere or have broader implications in rhizobiome community composition.

### Gibberellic acid – stimulant bridging microbial development and host colonisation

GAs regulate primary root elongation, increase the number of lateral roots (Dodd *et al*., 2010; Vanstraelen and Benková, 2012) and are thereby involved in the creation of microbial habitats (Figure 3). GAs were first isolated from the phytopathogenic fungus *Fusarium fujikuroi* (syn. *Gibberella fujikuroi*). Only a few bioactive GAs (e.g. GA1, GA3, GA4, GA7) affect plant growth and development, with major impacts on cell division and elongation, stem and root elongation, seed germination and flower and seed development (Yamaguchi, 2008). The typical symptoms of the ‘bakanae’ or ‘foolish seedlings’ disease of rice caused predominantly by *F. fujikuroi* are an excessive elongation and yellowing of diseased leaves, which can be directly attributed to the hormonal function of bioactive GAs produced by the fungus (Wulff *et al*., 2010; Jeon *et al*., 2013; Suga *et al*., 2019). Interestingly, although GA biosynthesis gene clusters are present in related plant‐colonising *Fusarium* spp., GA biosynthesis is not a general feature, but is limited to *F. fujikuroi* (Bömke and Tudzynski, 2009; Wiemann *et al*., 2013). The fact that a *F. fujikuroi* mutant lacking GA biosynthesis was strongly confined in its ability to invade rice cells points to a role of GAs as virulence effectors in *F. fujikuroi* (Wiemann *et al*., 2013). Whether the virulence of a *F. fujikuroi* strain is dependent on the amount of GAs it produces is still not entirely clear. The biosynthesis of bioactive GAs has been detected in a number of other plant pathogenic and non‐pathogenic fungi (e.g. *Phaeosphaeria* sp., *Aspergillus niger*, *Neurospora crassa*, *Penicillium* spp.) and in bacteria (e.g. some strains of *Acetobacter*, *Azospirillum*, *Bacillus*, *Bradyrhizobium* and *Rhizobium* spp.) (MacMillan, 2001; Bottini *et al*., 2004; Bömke and Tudzynski, 2009; Dodd *et al*., 2010; Khan *et al*., 2015; Spaepen, 2015; Tsukanova *et al*., 2017; Salazar‐Cerezo *et al*., 2018). Strikingly, the biosynthetic gene cluster for the GA precursor *ent*‐kaurene was identified to be significantly enriched in genomes of plant‐associated bacteria, suggesting that GA may support bacterial colonisation of plant environments (Levy *et al*., 2018a). GAs can support spore germination and hyphal growth in different fungal species, for example, *F. fujikuroi*, *Rhizophagus irregulare* and *Penicillium* spp., but do not seem to have a broader physiological function in fungi (Nakamura *et al*., 1988; Rademacher, 1994; Mercy *et al*., 2017). It is also unclear why some AMF produce GAs, as exogenous GA application inhibits AM formation, and GAs are generally considered to have a negative impact on this symbiosis (Barea and Azcón‐Aguilar, 1982; Bedini *et al*., 2018; Das and Gutjahr, 2020; Foo, 2020). As in other fungi, GAs may support hyphal growth of AMF. This is possibly supported by the observation that, although it inhibited arbuscule formation, treatment of *L. japonicus* with GA_3_ promoted hyphal growth and branching inside the root (Takeda *et al*., 2015). In addition to *F. fujikuroi*, several endophytic fungi can produce GAs in culture media. Some of these fungi transfer beneficial traits to their host plants, including growth promotion and increased stress tolerance (Khan *et al*., 2015). Whether these beneficial effects of endophytic fungi can be associated with their ability to produce GAs remains to be determined.

Rhizobia such as *B*. *japonicum*, *Sinorhizobium fredii* and *Rhizobium phaseoli* possess GA biosynthesis gene clusters, and can produce GAs (Atzorn *et al*., 1988; Boiero *et al*., 2007; Bano *et al*., 2010; Méndez *et al*., 2014; Nett *et al*., 2017). During nodulation, GAs inhibit initial colonisation events, but, likely due to their function in cell division and elongation, support nodule organogenesis (Foo, 2020). Although expression of rhizobial GA biosynthesis genes and the presence of presumably bacteria‐derived GAs was demonstrated in symbiotic bacteroids and nodules, GA operon knockouts in *B. japonicum* did not affect nodule formation and symbiosis, and therefore the physiological function of GA biosynthesis in rhizobia–plant interactions remains unclear (Tully and Keister, 1993; Rademacher, 1994; Nett *et al*., 2017). Interestingly, the bacterial leaf pathogen *Xanthomonas oryzae* pv. *oryzicola* possesses a GA biosynthesis operon, which is homologous to the one in rhizobia (Lu *et al*., 2015; Nett *et al*., 2017). The insertional disruption of genes within this operon led to reduced virulence of the bacterial pathogen on rice plants, and this was accompanied by an increased expression of marker genes for JA‐mediated defence. It has been suggested that *X. oryzae* pv. *oryzicola* likely produces a GA that can antagonise JA‐mediated defence, thus supporting bacterial virulence on its host plant.

PGPBs can produce bioactive forms of GA, or release them enzymatically from inactive conjugated forms (Bottini *et al*., 2004; Bömke and Tudzynski, 2009; Dodd *et al*., 2010; Glick, 2012; Spaepen, 2015). Low levels of available N stimulate GA production by bacteria in culture, while impaired gas exchange and osmotic stress reduce it (Bottini *et al*., 2004). Often an increase in root and/or shoot growth and germination can be observed after infection with GA‐producing PGPBs (Cassán *et al*., 2014; Shahzad *et al*., 2016). Inoculation with a GA‐producing *Bacillus amyloliquefaciens* increased growth of rice plants and suppressed endogenous levels of JA and ABA (Shahzad *et al*., 2016). Plants treated with GA‐producing bacteria can also be more tolerant to abiotic stress factors. Pepper (*Capsicum annuum*) plants inoculated with a GA‐producing strain of *Serratia nematodiphila* accumulated more GA and ABA under cold treatment (Kang *et al*., 2015). However, unless bacterial hormone biosynthesis mutants become available, it will be hard to evaluate which direct effects the microbial‐derived GAs have on plant growth.

### Abscisic acid – driver of the microbe–plant fitness alliance

The plant hormone ABA regulates developmental processes, is involved in biotic and abiotic stress responses, adjusts (root) growth and controls transpiration under stress conditions such as drought, high salinity or heavy metal toxicity (Ton *et al*., 2009; Cutler *et al*., 2010; Pieterse *et al*., 2012; Vishwakarma *et al*., 2017). Many plant colonising and saprophytic fungi can produce ABA. *Botrytis cinerea*, *M. oryzae*, *U. maydis*, *Verticillium* *dahliae* and *Alternaria brassicicola* are among the plant pathogenic fungi capable of ABA biosynthesis (Kettner and Dörffling, 1995; Siewers *et al*., 2006; Hartung, 2010; Bruce *et al*., 2011; Hauser *et al*., 2011; Spence and Bais, 2015; Spence *et al*., 2015; Shi *et al*., 2017; Han and Kahmann, 2019; Meents *et al*., 2019). ABA supports spore germination and fungal growth, and fungus‐derived ABA was required for appressorium formation and supported virulence of *M. oryzae* on rice plants (Spence and Bais, 2015; Spence *et al*., 2015; Chanclud and Morel, 2016). Exogenous ABA application at relatively low levels supports plant colonisation by AMF and arbuscule formation, while higher concentrations have negative effects on colonisation (Bedini *et al*., 2018; Das and Gutjahr, 2020; Foo, 2020). ABA treatment reduced the germination rate of spores of the AMF *Rhizoglomus irregulare*, but increased hyphal branching (Mercy *et al*., 2017). AMF not only increase endogenous levels of plant ABA during colonisation, they can also synthesise ABA, but it is unclear how much fungus‐derived ABA contributes to AM symbiosis (Esch *et al*., 1994; Chanclud and Morel, 2016; Bedini *et al*., 2018; Ludwig‐Müller, 2020). ABA production was also shown for some ectomycorrhizal fungi and some rhizobia, for example, *B*. *japonicum* (Boiero *et al*., 2007; Bano *et al*., 2010; Morrison *et al*., 2015). However, ABA is generally known to inhibit nodule formation, apparently by interfering with Nod factor signalling (Foo, 2020; Mathesius, 2020). Several rhizobacteria species can produce and secrete ABA in culture media, and some of these have been shown to increase ABA levels in plants (Forchetti *et al*., 2007; Sgroy *et al*., 2009; Dodd *et al*., 2010; Cohen *et al*., 2015; Tsukanova *et al*., 2017; Rosier *et al*., 2018). The physiological function of ABA in bacteria is unclear. Endophytic bacteria of sunflower (*Helianthus annuus*) produce more ABA (and JA) under osmotic stress. *Azospirillum* strains isolated from arid/semi‐arid or water‐stressed areas contained more ABA than those isolated from well‐watered areas, and wheat plants performed better under water stress when they were inoculated with the high‐ABA level *Azospirillum* strains (Forchetti *et al*., 2007; Ilyas and Bano, 2010). An ABA‐producing *Azospirillum* strain also increased Arabidopsis root length and ABA levels, even in an ABA biosynthesis mutant. Wild‐type plants inoculated with the ABA producer were also more tolerant to drought stress (Cohen *et al*., 2015). Conversely, some rhizobacteria possess the ability to degrade ABA (Hasegawa *et al*., 1984; Belimov *et al*., 2014). Two of these strains were shown to use ABA as a sole carbon source when grown in media, change root morphology and lower ABA levels in roots and/or shoots when applied to plants. However, the lack of bacterial mutants defective in ABA biosynthesis or metabolism makes it difficult to determine whether the changes in plant ABA levels and root morphology, as well as increased plant stress tolerance, are direct effects of bacterial ABA production or degradation (Dodd *et al*., 2010; Kudoyarova *et al*., 2019).

### Salicylic acid and jasmonic acid – targets for microbial manipulation of host communication

SA and JA are major regulators of mutually inhibiting defence signalling pathways against (hemi)biotrophic and necrotrophic pathogens, respectively (Glazebrook, 2005; Pieterse *et al*., 2012). SA and JA can also have direct negative effects on fungal or bacterial growth (Miersch *et al*., 1999; Hao *et al*., 2019). Many plant pathogens can interfere with either SA (e.g. *U. maydis*, *Phytophthora sojae*, *V. dahliae*) or JA (e.g. *M. oryzae*) accumulation or defence signalling in order to support virulence (Patkar *et al*., 2015; Lanver *et al*., 2017). Depending on its own lifestyle, a pathogen may make use of the negative cross‐talk between SA and JA signalling and activate one of the pathways in order to suppress the other. Several pathogen effectors have been identified, which interfere with SA or JA defence signalling components (Kazan and Lyons, 2014; Gimenez‐Ibanez *et al*., 2016; Han and Kahmann, 2019). Some pathogenic fungi or oomycetes can already prevent the formation of or degrade plant‐derived SA: *P*. *sojae* and *V. dahliae* secrete isochorismatases, which hydrolyse the SA precursor isochorismate, in order to suppress SA‐mediated defence responses (Liu *et al*., 2014). *Ustilago maydis* secretes both a chorismate mutase, which converts the SA precursor chorismate, and an SA hydroxylase, which converts SA into catechol (Djamei *et al*., 2011; Rabe *et al*., 2013). However, while the former enzyme is associated with lower SA levels inside the host plant and better colonisation, the latter can help the fungus to use SA as carbon source, albeit without any apparent virulence function. SA hydroxylases have been identified in other (phytopathogenic) fungi and soil bacteria (Rabe *et al*., 2013; Hao *et al*., 2019). Some pathogenic microbes can also produce SA, JA, JA conjugates or molecular mimics. SA has, for example, been detected in mycelia of the biotrophic fungal pathogen *V. dahliae* and the necrotroph *A. brassicicola* (Gimenez‐Ibanez *et al*., 2016; Meents *et al*., 2019). *Gibberella fujikuroi*, *M. oryzae*, some species of the genus *Lasiodiplodia* and certain ff. spp. of *F. oxysporum* are among the JA‐producing pathogenic fungi (Miersch *et al*., 1992; Cole *et al*., 2014; Gimenez‐Ibanez *et al*., 2016). At least the causal agent of the witch’s broom disease of cocoa (*Theobroma cacao*), *Moniliophthora perniciosa*, can even produce both, SA and JA (plus IAA and ABA), and both hormones supported fungal growth *in vitro* (Kilaru *et al*., 2007). However, it is not always clear how or to what extent the microbial production of SA and/or JA contributes to pathogen virulence (Thatcher *et al*., 2009; Cole *et al*., 2014; Chanclud and Morel, 2016). *Gibberella fujikuroi* and some ff. spp. of *F. oxysporum* can produce the bioactive JA conjugate JA‐Ile, which can bind to the COI1–JAZ co‐receptor to activate plant JA signalling, and may therefore alter JA signalling directly, to promote virulence (Cole *et al*., 2014; Kazan and Lyons, 2014). In a similar way, some strains of the bacterial plant pathogen *P. syringae* produce coronatine, which mimics JA‐Ile, and can therefore bind to the COI1–JAZ co‐receptor as well. Coronatine supports pathogen infection by activating JA signalling, thus re‐opening stomata for bacterial entry, suppressing SA‐mediated defence responses and promoting disease symptom development (Brooks *et al*., 2005; Melotto *et al*., 2006; Gimenez‐Ibanez *et al*., 2016; Kunkel and Harper, 2018). The grapevine (*Vitis vinifera*) pathogen *Lasiodiplodia mediterranea* produces an inactive JA ester, lasiojasmonate A, during the late stages of infection, and apparently lets the plant convert it into bioactive JA‐Ile in order to activate cell death‐related JA responses. This may support the fungus’ necrotrophic growth phase (Chini *et al*., 2018). In contrast, *M. oryzae* secretes a monooxygenase during plant colonisation, probably in order to convert fungus‐ and plant‐derived JA, thus avoiding activation of JA‐mediated defence responses (Patkar *et al*., 2015).

SA production has been detected in the beneficial root endophytic fungi *S. indica* and *Mortierella hyalina*, although it is not known whether this is required for plant colonisation or the beneficial effects on the host plants (Meents *et al*., 2019). The ectomycorrhizal fungus *Pisolithus tinctorius* can synthesise and metabolise JA, but it is not clear to what extent this affects mycorrhisation (Miersch *et al*., 1999).

PGPBs and plant growth‐promoting fungi can activate ISR against a broad spectrum of pathogens via SA‐ or JA (and ET)‐dependent signalling pathways and several (ISR‐inducing) PGPBs produce SA or JA (Forchetti *et al*., 2007; Van der Ent *et al*., 2009; Dodd *et al*., 2010; Bakker *et al*., 2014). Although in a few cases ISR activation has been attributed to some PGPBs’ ability to synthesise SA, bacterial SA biosynthesis does not seem to be a general requirement for it (De Meyer and Höfte, 1997; Maurhofer *et al*., 1998; De Meyer *et al*., 1999; Ran *et al*., 2005; Dodd *et al*., 2010; Bakker *et al*., 2014; Tsukanova *et al*., 2017; Rosier *et al*., 2018). Bacteria‐produced SA has also been discussed as a precursor for siderophores and its biosynthesis may support bacterial growth under iron‐limiting conditions (Bakker *et al*., 2014). Some antibacterial and antifungal activity has been attributed to SA and, furthermore, SA was shown to negatively affect biofilm formation and quorum sensing. These effects could impact the composition of soil microbial communities (Van Duy *et al*., 2007; Bakker *et al*., 2014; Lebeis *et al*., 2015). Conversely, SA degradation has been observed in several strains of the genera *Arthrobacter*, *Bacillus* and *Pseudomonas* (Dodd *et al*., 2010; Tsukanova *et al*., 2017). JA production has not been studied very intensively; however, JA (and ABA) production was detected in endophytic bacteria isolated from plants grown under drought stress, and it has been suggested that this may help plants to cope with stress conditions such as drought or salinity (Forchetti *et al*., 2007).

In summary, the ability to perceive, produce and/or degrade hormones is commonly found in a broad range of microbes. Depending on microbial lifestyles and niche preferences, the usage of hormone‐based communication serves at least two aims. In addition to enhancing their competitiveness in the rhizosphere by inhibiting growth or fitness of competing microbes, microbes employ hormones to prepare the plant endosphere for colonisation by adjusting root development and manipulating plant defence.

## CONCLUSION AND OUTLOOK

Plants and microbes have co‐evolved diverse strategies to communicate and thus interact with each other (Leach *et al*., 2017). From a plant perspective, it is a balancing act. Plants invest significant resources such as nutrients to create an attractive rhizosphere habitat for microbes. The merit to annex beneficial microbes and their beneficial activities comes with the risk of wasting costly resources to unhelpful or even detrimental microbes. It is therefore crucial for plants to have selective mechanisms in place to have some control over the access to the resource‐rich rhizosphere and to balance interactions. Hormones apparently take an essential role here and despite our limited knowledge seem to be as important for microbiome assembly as they are for the outcome of bilateral plant–microbe interactions.

For plants, hormones represent evolution‐driven, effective mediators to adapt to a diversity of environmental stimuli and to coordinate complex developmental processes (e.g. root system architecture). Considering the versatile roles of hormones, it is not surprising that microbes have adapted to and even hijacked hormone signalling. As a kind of common chemical language, hormones have evolved into go‐betweens of microbes and plants to implement conditions for the establishment of niches at the rhizosphere and to facilitate targeted host adaptation for endosphere colonisation. Astonishingly, microbes do not only synthesise hormones but perceive hormones to activate signalling processes to outcompete competitors and build alliances, suggesting the adoption of hormones in steering microbe–microbe interactions in a plant‐independent manner.

While the importance of hormones in the outcome of plant–microbe interactions is well known, we are just starting to understand the go‐between role of plant‐ as well as microbe‐derived hormones in root microbiome assembly. Our efforts to employ microbial communities, for instance for more sustainable and biodiversified crop production systems, require a better understanding of hormonal activities and their prevalence in plant–microbiome interactions. In this respect, it is essential to detect the origin of hormone synthesis and signalling (e.g. plant, tissue, cell type, microbe, species, rhizosphere, endosphere, etc.) and to determine their effect on plants and the assembly of microbial communities under different or changing environments. Recent advances in omics‐based technologies such as metatranscriptomics, metaproteomics or metabolomics (Levy *et al*., 2018a, 2018b,2018a, 2018b) combined with amplicon sequencing can help us in cataloguing hormonal processes in plant/root holobionts. Metabolomic profiling of root exudates will facilitate the identification of those compounds impacted by hormone signalling with a potential to shape the microbiome whilst also giving greater insight into the functional molar ranges of exuded plant‐derived and microbe‐derived hormones. These techniques are already being employed with some success (Dafoe *et al*., 2011; Badri *et al*., 2013; Chaparro *et al*., 2013; Zhalnina *et al*., 2018; Kudjordjie *et al*., 2019). These technologies do not replace amplicon sequencing but provide an additional, richer layer of information describing the broad functional nature of communities, not limited to only taxonomic classifications. To further assign hormone activities to individual members and to gain mechanistic insights requires functional approaches such as cell‐based assays with plants also lacking defined hormone synthesis and signalling components (Yoo *et al*., 2007; Lehmann *et al*., 2020). In addition to reductionist approaches with single microbes those functional analyses can be applied to characterise and assemble synthetic communities. Finally, we need to advance techniques to culture microbes and share knowledge and microbial strains (isolates) through existing resources and infrastructures. Culture collections curated by non‐profit biological resource centres can host valuable information (e.g. origin [soil type, plant association, etc.], activities, links to omics data, etc.) to support efforts to generate synthetic microbial communities and to validate their functionality in different ecosystems (e.g. as part of detoxification, renaturation, crop cultivation, etc.). Understanding the communication patterns of plants and microbes is of outstanding importance in this process to access the full genetic and ecological potential of microbiomes.

## CONFLICT OF INTEREST

The authors declare no conflict of interest.
